# Indoor Localization Based on Weighted Surfacing from Crowdsourced Samples

**DOI:** 10.3390/s18092990

**Published:** 2018-09-07

**Authors:** Junhong Lin, Bang Wang, Guang Yang, Mu Zhou

**Affiliations:** 1 School of Electronic Information and Communications, Huazhong University of Science and Technology (HUST), Wuhan 430074, China; junhonghust@hust.edu.cn (J.L.); guangyang@hust.edu.cn (G.Y.); 2 Chongqing Key Lab of Mobile Communications Technology, School of Communication and Information Engineering, Chongqing University of Posts and Telecommunications, Chongqing 400065, China; zhoumu@cqupt.edu.cn

**Keywords:** fingerprinting localization, sample crowdsourcing, sample weighting, surface fitting

## Abstract

Fingerprinting-based indoor localization suffers from its time-consuming and labor-intensive site survey. As a promising solution, sample crowdsourcing has been recently promoted to exploit casually collected samples for building offline fingerprint database. However, crowdsourced samples may be annotated with erroneous locations, which raises a serious question about whether they are reliable for database construction. In this paper, we propose a cross-domain cluster intersection algorithm to weight each sample reliability. We then select those samples with higher weight to construct radio propagation surfaces by fitting polynomial functions. Furthermore, we employ an entropy-like measure to weight constructed surfaces for quantifying their different subarea consistencies and location discriminations in online positioning. Field measurements and experiments show that the proposed scheme can achieve high localization accuracy by well dealing with the sample annotation error and nonuniform density challenges.

## 1. Introduction

Fingerprinting has been extensively researched for indoor localization systems in the last decade [1,2,3,4]. The basic idea is based on the assumption that each indoor location can be identified by a unique signal feature, called *fingerprint*. The widely used fingerprint is a vector of the *received signal strengths* (RSS) from the *access points* (AP) of wireless local access networks. The location of a test fingerprint can be estimated to a known location with minimal signal difference. One of the key challenges to support such fingerprinting localization is to construct an indoor *radio map* in the offline training phase [5,6,7,8]. Normally, the indoor environment is divided into non-overlapping grid cells. *Site survey* is often used to collect RSS samples at each grid center by surveyors for training one *grid fingerprint* for each grid. However, this scheme suffers from the time-consuming and labor-intensive site survey for radio map construction.

Recently, fingerprint crowdsourcing has been promoted to relieve or even eliminate the burdensome site survey by exploiting casually collected RSS samples [6,7,8,9]. Although not collected at specified locations, crowdsourced RSS samples still need to be annotated with some location information for fingerprint database construction. To this end, a common approach is to extract RSS samples from pedestrian movement trajectory [10,11,12]. As long as a trajectory can be correctly matched to one physical route, each step position can be obtained from the floor plan to annotate the corresponding step RSS sample.

Although fingerprint crowdsourcing seems a promising approach, care must be taken to deal with the samples with erroneously annotated locations. Compared with the site survey, such erroneous location annotation of crowdsourced samples could lead to an inaccurate radio map and degrade the performance of fingerprinting-based localization. Besides annotation errors, another challenge lies in that crowdsourced samples may not be uniformly distributed in the whole environment.

In this paper, we study the indoor localization through constructing radio propagation surfaces from crowdsourced samples. For each AP, its surface takes a location as input and outputs an estimated RSS of this location. To deal with annotation errors, we propose a *cross-domain cluster intersection* algorithm to assign each sample a reliability weight, which exploits the sample clustering results from both the physical and signal space. We next select a subset of weighted samples to fit each AP a surface from polynomial primary functions and construct subarea fingerprints by sampling AP surfaces. Furthermore, we compute two weights for each AP surface for describing its subarea consistency and location discrimination capability in online positioning. A two-step positioning algorithm is proposed to first determine the belonging subarea for a test sample, and then a weighted surface search is exploited to estimate its location within the subarea. We conducted field measurements and experiments. Compared with the peer schemes, results validate the effectiveness and robustness of the proposed scheme in terms of its lower localization error when facing sample annotation error and nonuniform density challenges.

The rest of the paper is organized as follows. Section 2 briefly reviews the most related work as well as the proposed system. The proposed offline surface fitting algorithm is presented in Section 3. Section 4 presents our online localization algorithm. Field measures are used for experiments and the results are provided in Section 5. Finally, Section 6 concludes the paper.

## 2. Related Work and System Overview

### 2.1. Related Work

Several fingerprinting systems based on sample crowdsourcing have been proposed for indoor localization in previous studies [13,14,15,16,17,18]. For example, Chen and Wang [13] proposed using a density-based clustering technique to group crowdsourced samples to generate a cluster fingerprint and using a matching algorithm to assign each cluster fingerprint to one subarea for room-level localization. Liu et al. [14] also applied crowdsourced samples for room-level localization yet with an improved energy-efficient sampling approach. Chang et al. [15] applied a local Gaussian process to construct grid fingerprints from crowdsourced samples. Jung et al. [16] adopted a hybrid global-local optimization scheme to determine the location of fingerprint sequences based on the constraint of the indoor structure, rather than using labeled fingerprints. They also proposed an unsupervised learning method to calibrate the localization model.

In the literature, many have proposed to extract crowdsourced samples from pedestrian trajectories. The core idea is to match a trajectory to one physical route such that each sample on a trajectory can be labeled with one location in the route [19,20,21,22,23,24,25]. For example, Kim et al. [19] combined the lightweight site survey and fingerprint crowdsourcing for radio map construction. They first constructed an initial radio map according to the lightweight site survey and use the *pedestrian dead reckoning* (PDR) to match the the war-walking paths into the radio map. Huang et al. [20] exploited layout landmarks such as the cross points of corridors for matching pedestrian trajectories to physical routes. Zhou et al. [23] proposed to transform the indoor layout into a semantic graph to map with activity sequences contained within the trajectories. Zhou et al. [25] applied a density-based spatial clustering algorithm to determine hotspots which are then mapped to physical subareas.

For crowdsourced samples, the conventional approach is to construct a radio map for grid fingerprints. In Ref. [26], Wang et al. proposed using polynomial functionals to fit a propagation surface for each AP based on a few reference fingerprints with correct location annotations. Ye and Wang [27] applied the surfacing method to deal with the problem of non-uniformly distributed crowdsourced samples, with the objective of composing grid fingerprints for radio map construction. Unlike these approaches, this paper proposes to exploit crowdsourced samples for fitting radio propagation surfaces. As crowdsourced samples normally have inaccurate location labels, how to construct a reliable surface is rather challenging. In this paper, we propose a sample weighting algorithm and apply weighted samples to fitting surfaces.

### 2.2. System Overview

We divided an indoor environment into several distinct subareas, such as rooms, corridors, etc., according to their functional layout by inherent obstructions and partitions such as concrete walls. We assumed that each crowdsourced sample has been annotated with some location, though possibly with annotation errors. We attributed each sample to one subarea according to its annotated location. The proposed system also consists of the offline and online phases.

The offline phase consists of four steps: Weighting crowdsourced samples assigns each crowdsourced sample a *reliability weight* based on our proposed *cross-domain cluster intersection* algorithm. Fitting radio surfaces constructs a radio propagation surface for each AP based on the weighted samples. Weighting fitted surfaces further assigns each fitted surface with two weights for discriminating their contributions for online localization. Constructing subarea fingerprints creates an RSS fingerprint for each subarea from its fitted and weighted surfaces.

**Figure 1:**
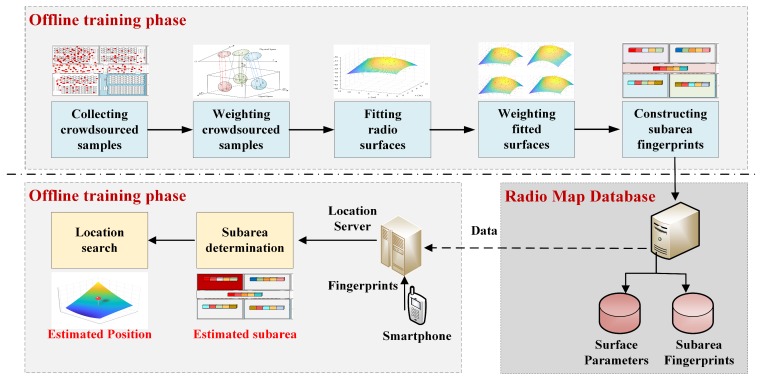
The flowchart of the proposed system: In the offline phase, crowdsourced samples are each weighted according to our algorithm. For each access point and for one subarea, its radio propagation surface is firstly fitted and also weighted from those selected and weighted samples. Subarea fingerprints are then composed from fitted surfaces. In the online phase, a test sample is first compared with subarea fingerprints to determine its belonging subarea, and then a gradient search is used to estimate its exact location.

The online localization consists of two steps: Subarea determination first locates an online test fingerprint into one subarea according to our proposed weighted signal distance. Location search searches the coordinate for the test fingerprint based on the gradient search on the constructed surfaces. Figure 1 presents the main flowchart of the proposed system, and Table 1 lists the symbols used in this paper as well as their notations.

## 3. The Offline Weighted Surfacing Algorithm

### 3.1. Weighting Crowdsourced Samples

In one subarea, e.g., a room, let $S=\{s_{i},\ldots ,s_{M}\}$ denote its set of *M* crowdsourced samples. A sample $s_{i}=\left({\vec{l}}_{i},{\vec{r}}_{i}\right)$ consists of two parts: ${\vec{l}}_{i}=\left(x_{i},y_{i}\right)$ is its annotated location; and ${\vec{r}}_{i}=\left(r_{i1},r_{i2},\ldots r_{iN}\right)$ the received RSS vector where *N* is the maximum number of hearable APs in one subarea. For one sample $s_{i}$, it is possible that not all the *N* APs could be heard, that is, some $r_{ij}$ (j<N) might not be available in $s_{i}$. In this case, to allow the clustering and surfacing algorithm to run normally, we simply set it to a very small RSS value, $r_{min}=-90$ dBm, which is the lower bound of the collected signal strength, during the following sample clustering and weighting process.

A crowdsourced sample $s_{i}$ may not be reliable in that its annotated location ${\vec{l}}_{i}$, RSS measurement ${\vec{r}}_{i}$, or both might have some errors. However, among a large number of such samples, we conjecture that some statistical relations could be extracted from the similarities between the physical and signal space. Consider the following example of two samples $s_{i}$ and $s_{j}$. Let $d_{ij}^{p}≜∥{\vec{l}}_{i}-{\vec{l}}_{j}∥$ and $d_{ij}^{s}≜∥{\vec{r}}_{i}-{\vec{r}}_{j}∥$ denote the distance between the two samples in the physical space and signal space, respectively. Suppose that $d_{ij}^{p}$ is small, indicating that $s_{i}$ and $s_{j}$ are close to each other according to their annotated locations. For a small $d_{ij}^{s}$, we could conjecture that both samples are reliable or both samples are unreliable. Although we could not determine which is the real case for only two samples, we might be able to infer the statistic relations from a large number of samples to discriminate unreliable samples. Motivated from such considerations, we next present a *cross-domain cluster intersection* (CCI) algorithm to assign each sample a *reliability weight*.

In both the physical and signal space, we group all samples $s_{i}\in S$ into *K* clusters by the classic *K-means* clustering algorithm. Let $C^{p}=\left\{C_{1}^{p},\ldots ,C_{K}^{p}\right\}$ and $C^{s}=\left\{C_{1}^{s},\ldots ,C_{K}^{s}\right\}$ denote the set of clusters in the physical and signal space, respectively. Notice that a sample $s_{i}$ is within one of the clusters in $C^{p}$ and $C^{s}$ simultaneously. We define a *cross-domain cluster coefficient* for such a sample $s_{i}$ based on the cluster intersection between $C_{a}^{p}$ and $C_{b}^{s}$ as follows:

(1) $$\gamma_{i}=\frac{\;|C_{a}^{p}⋂C_{b}^{s}{|}^{2}}{|C_{a}^{p}\left|\times \right|C_{b}^{s}|}.$$

**Figure 2:**
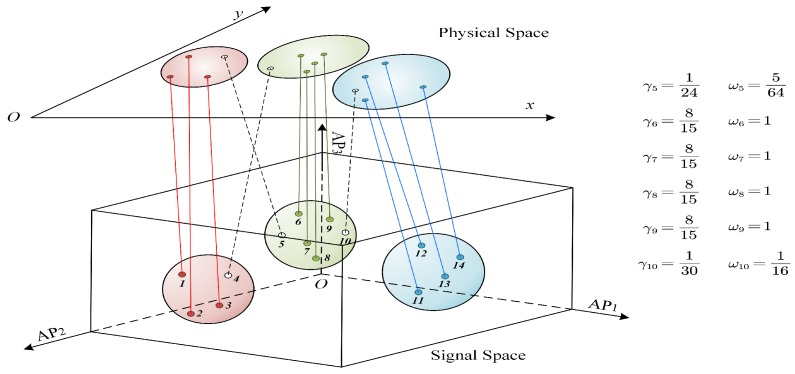
Illustration of the cross-domain cluster intersection algorithm: In the physical space, samples are clustered according to their annotated coordinates. In the signal space, samples are clustered according to the RSS distances. The weight of a sample is determined by the common samples between its belonged physical cluster and signal cluster.

If $C_{a}^{p}=C_{b}^{s}$, i.e., the two clusters contain the same set of samples, then all such samples have the same coefficient and $\gamma_{i}=1$. According to the *K-means* clustering, all samples in $C_{a}^{p}$ are closer to this cluster center than to other cluster centers. This is also the case for samples in $C_{b}^{s}$ in the signal space in terms of their RSS vector similarities. Therefore, $|C_{a}^{p}⋂C_{b}^{s}|$ describes how many samples are close to each other in both the physical and signal space. A small value of $\gamma_{i}$ indicates that $s_{i}$ is not similar to the majority of the two clusters, which might suggest its unreliability. As the surface fitting is done in the signal space, we further normalize $\gamma_{i}$ to assign the sample weight based on the signal space clusters. For each sample $s_{i}\in C_{b}^{s}$, we compute its reliability weight by

(2) $$\omega_{i}=\frac{\gamma_{i}}{\max\{\gamma_{j}|s_{j}\in C_{b}^{s}\}},$$

where the denominator is the maximum cross-domain cluster coefficient of the samples in the cluster. Figure 2 illustrates the CCI algorithm and computes reliability weights for some samples.

### 3.2. Fitting Radio Surfaces

In one subarea, we construct a radio propagation surface for each hearable AP based on the weighted samples $\left(s_{i},w_{i}\right)$. A surface function ϕ(x,y) takes a location as its input and outputs an estimated RSS at this location. Notice that the number of crowdsourced samples could be large and keep increasing. To reduce computational complexity and alleviate surface overfitting, we propose a *percentile weight partition* (PWP) method to select only a subset of weighted samples.

Define $p_{th}$ and $\omega_{th}$ as the percentile and weight threshold, respectively, and $p_{th},\omega_{th}\in \left[0,1\right]$. The objective is to ensure that more than $p_{th}$ samples have weights larger than $\omega_{th}$. We first sort samples according to their weights in an increasing order, denoted by $\vec{\omega }$. Let $\omega_{k}$ denote the *k*th sample whose weight is at the $p_{th}$ percentile of $\vec{\omega }$. If $\omega_{k}\geq \omega_{th}$, then no samples will be removed. Otherwise, we remove the first $\left\lceil \frac{M-k\omega_{th}}{1-\omega_{th}}\right\rceil$ samples from $\vec{w}$. After the sample selection, let $S^{\prime }$ denote the set of select samples and let A denote the set of hearable APs by samples in $S^{\prime }$.

In this paper, we adopt a polynomial function to fit a radio propagation surface for each AP in A as follows:

(3) $$\phi_{n}\left(x,y\right)=\sum_{i=1}^{p}\sum_{j=1}^{q}a_{ij}x^{i-1}y^{j-1},\;\mathrm{for}\;\mathrm{all}\;n\in A,$$

where $a_{ij}$s are fitting coefficients. The objective of weighted surface fitting is to

(4) $$\mathrm{minimize}\;H\equiv \sum_{i=1}^{|S^{\prime }|}\omega_{i}^{2}{\left(\phi_{n}\left(x_{i},y_{i}\right)-r_{in}\right)}^{2}$$

To compute one fitting coefficient $a_{er}$, we equate its partial derivative to zero to minimize *H*.

(5) $$\begin{array}{cc} \frac{\partial H}{\partial a_{er}} & =\frac{\partial }{\partial a_{er}}\sum_{i=1}^{n}\omega_{i}^{2}{\left[\phi_{n}\left(x_{i},y_{i}\right)-r_{in}\right]}^{2}\\ & =\sum_{i=1}^{n}\left\{2\omega_{i}^{2}\left[\phi_{n}\left(x_{i},y_{i}\right)-r_{in}\right]\frac{\partial }{\partial a_{er}}\left[\phi \left(x_{i},y_{i}\right)\right]\right\}\\ & =\sum_{i=1}^{n}\left\{2\omega_{i}^{2}\left[\phi_{n}\left(x_{i},y_{i}\right)-r_{in}\right]x_{i}^{e-1}y_{i}^{r-1}\right\}\\ & =0 \end{array}$$

From the equation above, we can derive

(6) $$\begin{array}{c} \sum_{i=1}^{n}2\omega_{i}^{2}x_{i}^{e-1}y_{i}^{r-1}\phi_{n}\left(x_{i},y_{i}\right)=\sum_{i=1}^{n}2\omega_{i}^{2}x_{i}^{e-1}y_{i}^{r-1}r_{in} \end{array}$$

(7) $$\begin{array}{c} \sum_{i=1}^{n}2\omega_{i}^{2}x_{i}^{e-1}y_{i}^{r-1}\sum_{c=1}^{p}\sum_{d=1}^{q}a_{cd}x_{i}^{c-1}y_{i}^{d-1}=\sum_{i=1}^{n}2\omega_{i}^{2}x_{i}^{e-1}y_{i}^{r-1}r_{in} \end{array}$$

We define

(8) $$\begin{array}{cc} u_{cd}\left(e,r\right) & =\sum_{i=1}^{n}\left(2\omega_{i}^{2}x_{i}^{c-1}y_{i}^{d-1}x_{i}^{e-1}y_{i}^{r-1}\right) \end{array}$$

(9) $$\begin{array}{cc} v\left(e,r\right) & =\sum_{i=1}^{n}2\omega_{i}^{2}x_{i}^{e-1}y_{i}^{r-1}r_{in} \end{array}$$

Thus, we can rewrite the equation as:

(10) $$\sum_{c=1}^{p}\sum_{d=1}^{q}a_{cd}u_{cd}\left(e,r\right)=v\left(e,r\right),\;e=1,\cdots ,p,\;r=1,\cdots ,q$$

The matrix form of equation above is:

(11) $$\left[\begin{array}{ccc} u_{11}\left(1,1\right) & \cdots & u_{pq}\left(1,1\right)\\ \vdots & \ddots & \vdots \\ u_{11}\left(p,q\right) & \cdots & u_{pq}\left(p,q\right) \end{array}\right]\left[\begin{array}{c} a_{11}\\ \vdots \\ a_{pq} \end{array}\right]=\left[\begin{array}{c} v\left(1,1\right)\\ \vdots \\ v\left(p,q\right) \end{array}\right]$$

Then, by $A=U^{-1}V$, the surface coefficient can be calculated.

### 3.3. Weighting Fitted Surfaces

Each AP surface is constructed based on its weighted samples. Different AP surfaces could contribute differently for describing the whole signal space. We next assign two weights to each AP surface via an entropy-like quantity computed from its samples: one is used for subarea determination and the other for location search in our online positioning.

For each AP in A, let $R=\{r_{1},\ldots ,r_{R}\}$ denote its set of RSS values extracted from the weighted samples in $S^{\prime }$. As the samples are assumed to be crowdsourced randomly from different locations, the set R is also expected to contain the RSS values from different locations. If all elements in R have similar values, then this AP might not be very helpful for discriminating different locations in one subarea. On the other hand, such an AP may be seen as a good indication of this subarea for its RSS consistency. Motivated by such considerations, we propose to weight AP surfaces for their different *subarea consistencies* and *location discriminations* from an entropy-like viewpoint.

We first normalize the elements in R by

(12) $${\bar{r}}_{i}=\frac{r_{i}-\min\left(R\right)}{\max(R)-\min(R)},\;\mathrm{for}\;\mathrm{all}\;r_{i}\in R.$$

We next compute an entropy-like quantity η for each AP in A to describe its RSS distribution property by

(13) $$\eta =-\frac{\sum_{i=1}^{R}p_{i}\ln\left(p_{i}\right)}{\ln(R)},\;\mathrm{where}\;p_{i}=\frac{{\bar{r}}_{i}}{\sum_{j=1}^{R}{\bar{r}}_{j}}.$$

For our two-step online positioning, we compute two surface weights for each AP:

(14) $$\rho_{n}^{sub}=\frac{\eta_{n}}{\sum_{j=1}^{\left|A\right|}\eta_{j}},\;\rho_{n}^{loc}=\frac{1-\eta_{n}}{\sum_{j=1}^{\left|A\right|}\left(1-\eta_{j}\right)}.$$

$\rho_{n}^{sub}$ is used in the subarea determination, while $\rho_{n}^{loc}$ is used in the location search in one subarea.

### 3.4. Constructing Subarea Fingerprints

For each subarea, we construct a *subarea fingerprint* $\vec{f}$ based on its weighted surfaces $\phi_{n}$ (n∈A). We adopt a grid lattice approach to sample each surface $\phi_{n}$ uniformly in the physical space. Let G denote such a grid structure. For the *g*th grid, let $f_{gn}=\phi_{n}\left(g_{x},g_{y}\right)$ denote a sampled grid RSS value from the *n*th surface, where $\left(g_{x},g_{y}\right)$ is the coordinate of the grid center. Then, $\vec{f}$ consists of subarea-averaged RSS values for all hearable APs

(15) $$\vec{f}=\left(\frac{1}{\left|G\right|}\sum_{g\in G}f_{gn},\ldots ,\frac{1}{\left|G\right|}\sum_{g\in G}f_{gN^{\prime }}\right),$$

where $N^{\prime }=\left|A\right|$ is the number of hearable APs in A.

## 4. The Online Positioning Algorithm

The online positioning consists of two phases: subarea determination and location search.

**Subarea Determination:** Let ${\vec{f}}_{t}$ denote the RSS vector of a test sample, and ${\vec{f}}_{s}$ the *s*th subarea fingerprint. Let $A_{int}$ denote the set of hearable APs by both ${\vec{f}}_{t}$ and ${\vec{f}}_{s}$. We compute the weighted signal distance between ${\vec{f}}_{t}$ and ${\vec{f}}_{s}$ as:

(16) $$D_{s}=\frac{1}{|A_{int}|}\sqrt{\sum_{n\in |A_{int}|}{\left(\rho_{n}^{sub}\times \left(f_{sn}-f_{tn}\right)\right)}^{2}},$$

where $f_{sn}$ and $f_{tn}$ are the RSS values from the *n*th hearable AP in ${\vec{f}}_{s}$ and ${\vec{f}}_{t}$, respectively. The test sample is then localized into a subarea with the minimum $D_{s}$.

**Location Search:** Assume that the *s*th subarea is selected in the first phase. We next search a space point $\left(\hat{x},\hat{y}\right)$ in this subarea to minimize the weighted signal difference between ${\vec{f}}_{t}$ and subarea surfaces:

(17) $$\left(\hat{x},\hat{y}\right)=\arg\min_{\left(x,y\right)}\sum_{n\in A_{int}}{\left[\rho_{n}^{loc}\left(\phi_{n}\left(x,y\right)-f_{tn}\right)\right]}^{2}$$

In this paper, we use the gradient descent search method. Instead of randomly choosing a start point, we use the localization result of a simple *nearest neighbor* (NN) algorithm as the initial searching point, where the grid fingerprints are spatially sampled from the fitted surfaces. We then calculate weighted signal difference as the cost function and its partial derivation to determine the search direction. The cost function is defined as

(18) $$J\left(l_{t}\right)=\sum_{n\in A_{int}}{\left[\rho_{n}^{loc}\left(\phi_{n}\left(x,y\right)-f_{tn}\right)\right]}^{2}$$

The search iteration is defined by

(19) $$\begin{array}{c} l_{t+1}=l_{t}+\alpha_{d}d_{t},\;\mathrm{where}\;d_{t}=-\nabla J\left(l_{t}\right) \end{array}$$

(20) $$\begin{array}{c} \nabla J\left(l_{t}\right)={\left[\frac{\partial J\left(l_{t}\right)}{\partial x},\frac{\partial J\left(l_{t}\right)}{\partial y}\right]}^{T}, \end{array}$$

where $\alpha_{d}$ is the search step. We substitute Equation (3) into Equation (18):

(21) $$J\left(l_{t}\right)=\sum_{n\in A_{int}}{\left[\rho_{n}^{loc}\left(\sum_{i=1}^{p}\sum_{j=1}^{q}a_{ij}x^{i-1}y^{j-1}-f_{tn}\right)\right]}^{2}$$

Next, we compute the partial derivation of this cost function to gain the gradient and update the search iteration.

(22) $$\begin{array}{cc} \frac{\partial J\left(l_{t}\right)}{\partial x}= & \sum_{n\in A_{int}}2{\left(\rho_{n}^{loc}\right)}^{2}\left[\phi_{n}\left(x,y\right)-f_{tn}^{0}\right]\sum_{i=1}^{p}\sum_{j=1}^{q}a_{ij}\left(i-1\right)\;\\ & x^{j-2}y^{j-1} \end{array}$$

(23) $$\begin{array}{cc} \frac{\partial J\left(l_{t}\right)}{\partial y}= & \sum_{n\in A_{int}}2{\left(\rho_{n}^{loc}\right)}^{2}\left[\phi_{n}\left(x,y\right)-f_{tn}^{0}\right]\sum_{i=1}^{p}\sum_{j=1}^{q}a_{ij}x^{i-1}\;\\ & \left(j-1\right)y^{j-2} \end{array}$$

The gradient search will stop when the $d_{t}$ is too small to update the search position for the next iteration.

## 5. Field Measurements and Experiments

### 5.1. Experiment Settings

**Figure 3:**
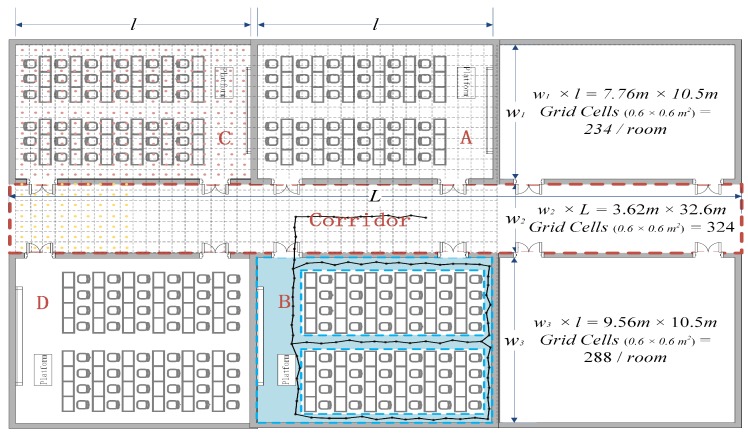
The layout of the indoor environment. A grid lattice has been used to collect samples, with in total 1368 grid cells each with size 0.6×0.6 m${}^{2}$. Besides, pedestrian trajectories have also been used to collect samples for the corridor and walkable pathways in each room.

Figure 3 plots the indoor layout of our field measurements in a typical lecture building with total area of 482 m${}^{2}$. In our work, we did not place our own APs. Instead, we employed the existing Wi-Fi infrastructure with APs deployed by different parties, such as individual laboratories, telecom operators and campus authorities. Indeed, the total number of hearable AP in our experimental environment was more than 400, while, for each sample, normally >70 APs could be heard. We note that emplying the existing Wi-Fi infrastructure makes our proposed scheme ready to be implemented in many practical scenarios.

A Huawei Honor 3C smartphone was used to collect RSS samples. We conducted two batches of sample collection: The first batch $S_{site}$ was based on the site survey approach, containing in total 13,670 samples each collected at one grid center. The second batch $S_{walk}$, containing in total 13,370 samples, was extracted from movement trajectories restricted to only those walkable routes, as illustrated by the colored area in one room in Figure 3. Note that the samples in both $S_{site}$ and $S_{walk}$ are firstly annotated true location information at collection. To emulate annotation errors, we again annotate each sample into a new location with a *location offset* randomly drawn from a Gaussian distribution with zero mean and σ standard deviation. The test set $S_{test}$ contains 5600 samples uniformly distributed in the whole environment.

**Experiment Schemes**: According to their annotated locations, crowdsourced samples can be assigned into different grids to construct grid fingerprints. Similarly, they can also be grouped into different clusters in the signal space to obtain cluster fingerprints. We tested the following peer localization schemes to examine these typical approaches.

- FGrid emulates the traditional site-survey fingerprinting based on grid fingerprints, which divides the subarea into several non-overlapping grid cell to contain samples, and assigns each new sample into its nearest grid cell. For each grid cell, a *grid fingerprint* is composed by averaging all samples located within the grid cell, and the location of the grid fingerprint is annotated as the grid center. In the online phase, we used the *nearest neighbor* algorithm.
- SGrid is similar to the FGrid to obtain grid fingerprints. We then constructed surfaces based on these fingerprints in the offline phase. In the online phase, we used the same surface search method as the one in our proposed SWSample.
- SRaw retains the original position of every crowdsourced sample and fits propagation surfaces based on them. In the online phase, we used the same surface search method as the one in our proposed SWSample.
- SCluster clusters the samples in signal domain only. For each cluster, we obtained a *cluster fingerprint*, which is the average of its cluster members’ RSS vectors. The location of a cluster fingerprint is the geometric center of the cluster members. We fitted the propagation surfaces for every AP based on these cluster fingerprints. In the online phase, we used the surface search method the same as the one in our proposed SWSample.
- SWSample is the proposed scheme.

In all the above schemes, we set the cluster number equal to the number of grids used in *FGrid* for a fair comparison. We also adopted the proposed two-step online positioning algorithm. We noticed that, from our experiments, the *subarea hitting rate* of all these schemes is not smaller than 99.58%, i.e., almost all test samples can be correctly determined to its belonging subarea. Thus, we do not report this result again in the following.

### 5.2. Surface Fitting Examples

**Figure 4:**
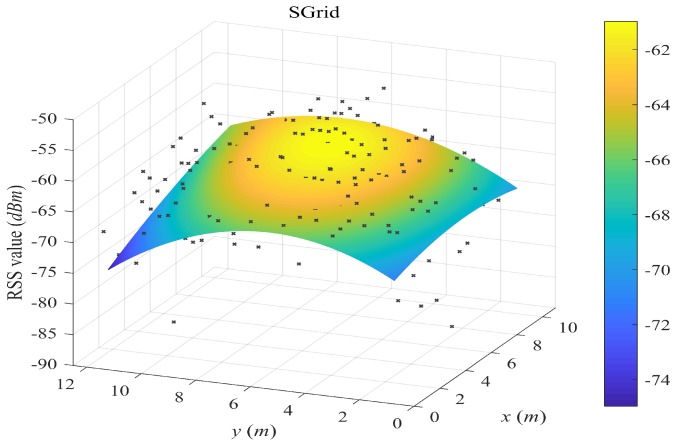
Illustration of fitted surface by SGrid. We choose one AP for Room A and fit its surface from 1800 samples randomly drawn from $S_{site}⋃S_{walk}$. Crowdsourced samples are assigned to grid cells. A grid fingerprint is composed by averaging all samples in the grid cell, and its location is the grid center. The fitted surface is based on the grid fingerprints.

**Figure 5:**
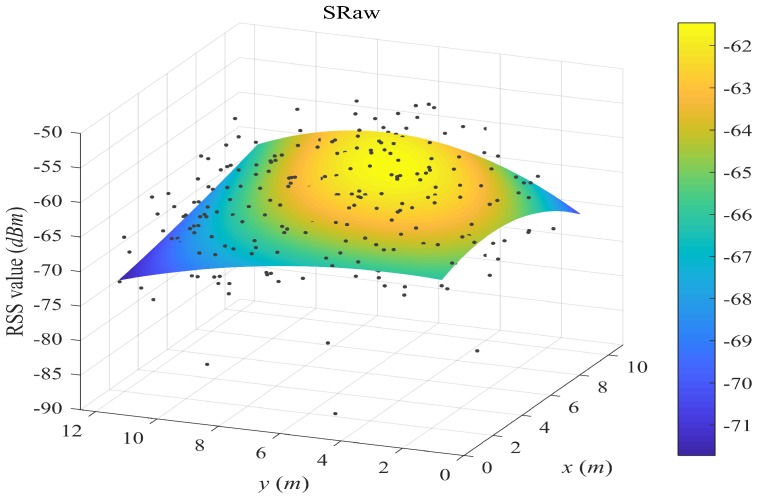
Illustration of fitted surface by SRaw. We choose one AP for Room A and fit its surface from 1800 samples randomly drawn from $S_{site}⋃S_{walk}$. All crowdsourced samples are used for surface fitting, without sample weighting and selection.

**Figure 6:**
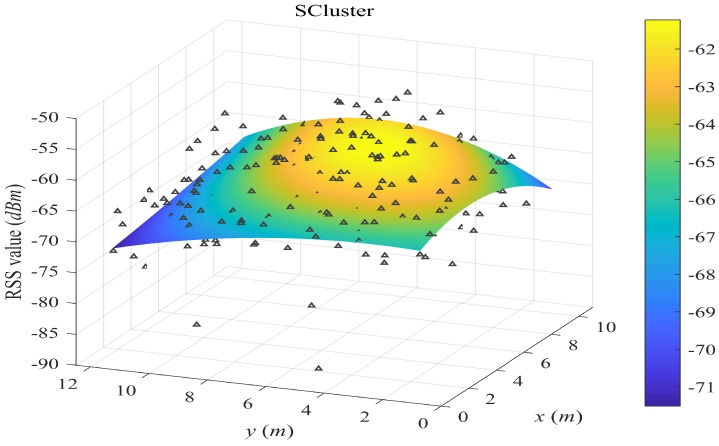
Illustration of fitted surface by SCluster. We choose one AP for Room A and fit its surface from 1800 samples randomly drawn from $S_{site}⋃S_{walk}$. All crowdsourced samples are first clustered in the signal space. For each cluster, a cluster fingerprint is composed by averaging the RSS vectors of its cluster members, and its location is the geometric center of the cluster members. The fitted surface is based on the cluster fingerprints.

**Figure 7:**
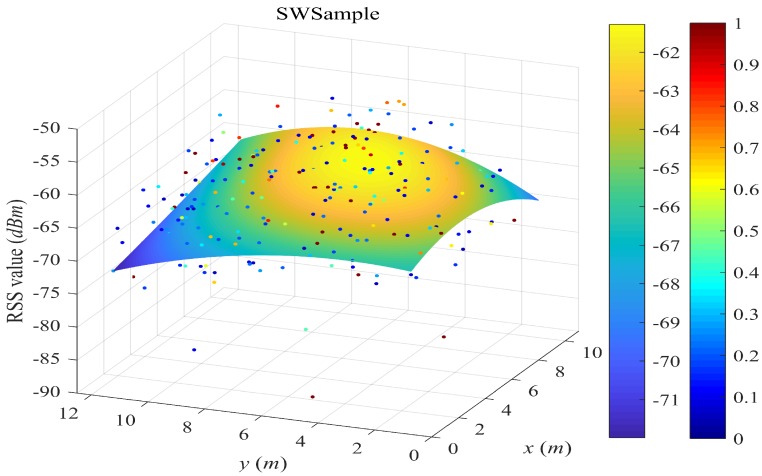
Illustration of fitted surface by our proposed SWSample. We choose one AP for Room A and fit its surface from 1800 samples randomly drawn from $S_{site}⋃S_{walk}$. Crowdsourced samples are weighted and selected for surface construction. The sample weight is illustrated by the dot color in the figure.

Figure 4, Figure 5, Figure 6 and Figure 7 plot the fitted surfaces for the four surfacing-based schemes. We chose one AP for Room A and fit its surface from 1800 samples randomly drawn from $S_{site}⋃S_{walk}$. For the SWSample fitted surface in Figure 7, we also color the sample weight as shown by the weight color spectrum alongside the graph. It can be seen that the proposed scheme could produce a smoother surface, compared with other schemes. If we assume that this AP is located at the coordinate around the highest RSS value, then we could observe that the surface in Figure 7 is more like an attenuated sphere centered at the AP. The Keenan–Motley path loss model has been widely adopted to characterize the radio propagation in mobile cellular networks. If such a model could still be applicable in a small and open space such as a room, then our fitted surface resembles the most to this model, which might also help to explain the effectiveness of our weighted surface fitting.

### 5.3. Experiment Results

**Figure 8:**
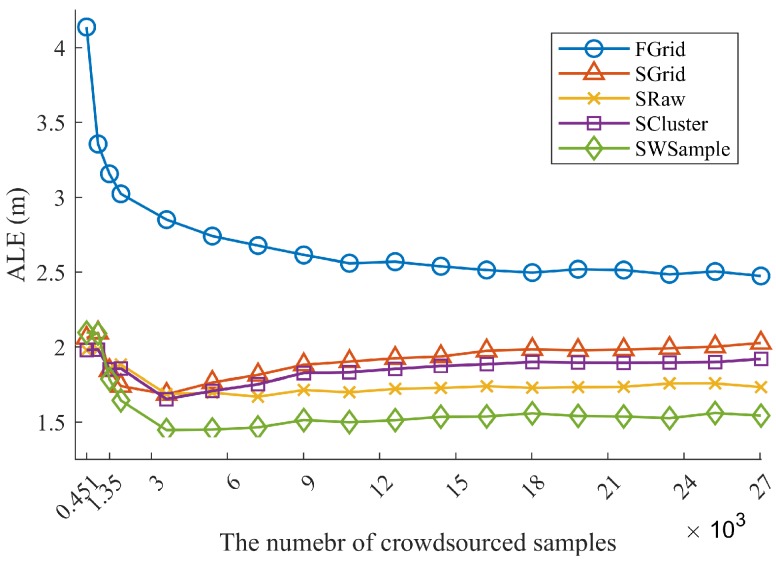
Comparison of localization performance. The average localization error (ALE) vs. the number of crowdsourced samples $M_{all}$, when using crowdsourced samples from $S_{site}⋃S_{walk}$. The standard deviation of location offset σ=1.2 m.

**Uniformly distributed samples:** We first considered the scenario that all crowdsourced samples are uniformly distributed in the experiment environment, that is, we used crowdsourced samples from $S_{site}⋃S_{walk}$. Figure 8 plots the *average localization error* (ALE) against the number of crowdsourced samples randomly drawn from $S_{site}⋃S_{walk}$. It was first observed that all the surfacing schemes outperform the grid fingerprinting FGrid, which validates the effectiveness of using fitted radio propagation surfaces for localization. When the number of samples increases, from about $0.33M_{g}$ to $20M_{g}$ with $M_{g}$ the number of total grid cells, the ALE of the surfacing schemes first decreases and then increases. At first, the number of samples is not large enough to well fit actual surfaces. In this case, our scheme SWSample has a slightly higher ALE than other surfacing schemes (see the first two points in Figure 8) due to its sample selection. On the other hand, if the noisy samples are too many, the surfaces may be overfitted for unreliable samples. However, ours presents a decent degradation and the ALE of using all 27,040 samples is 1.54 m, slightly higher than the best case of 1.45 m of using 3605 samples. The positioning accuracy improvement of our scheme are 36.71% over FGrid and 9.41% over SRaw, respectively. Compared with the SRaw scheme, the improvement can be attributed to our sample weighting and selection algorithm, which only chooses those reliable samples for weighted surface fitting, leading to a more accurate radio map and better positioning results.

**Figure 9:**
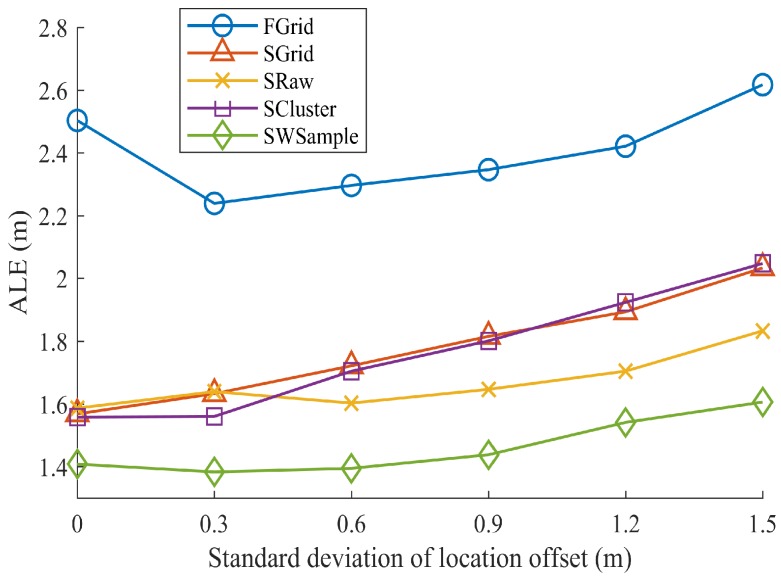
Comparison of localization performance. The average localization error (ALE) vs. the standard deviation σ of location offset, where $M_{all}=27,040$.

**Figure 10:**
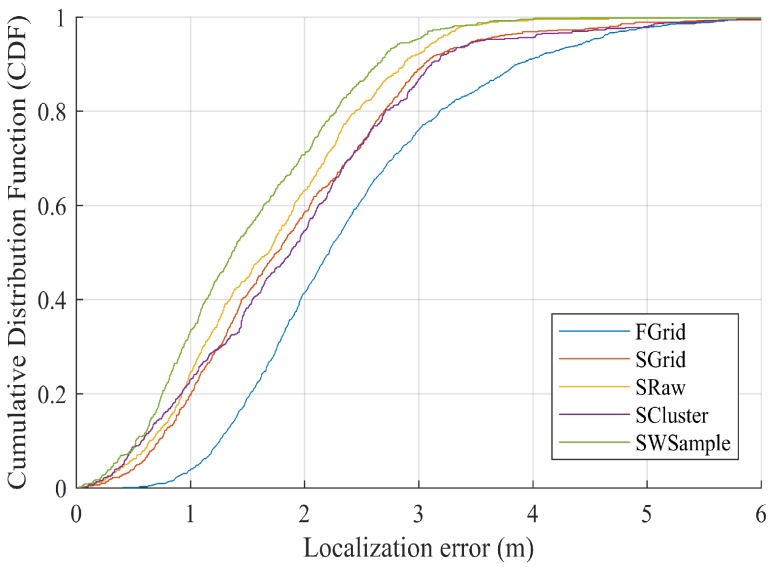
Comparison of cumulative distribution function (CDF) localization error, where $M_{all}$ = 27,040 and σ=1.2 m.

Figure 9 presents the ALE against the standard deviation σ of location offset. Notice that σ=0 indicating no annotation errors. It is not unexpected to see that all schemes suffer from the increasing of σ, i.e., the annotated locations farther away from true locations. However, our scheme SWSample still performs the best. Figure 10 plots the *cumulative distribution function* (CDF) of localization error. It is worth noting that, besides a low median localization error of 1.51 m, our SWSample has a low 90% percentile error of only 2.64 m. To provide the exact numbers, Table 2 summarizes the localization error results for three situations, namely, σ=0.6 m, σ=0.9 m, and σ=1.2 m, respectively.

**Figure 11:**
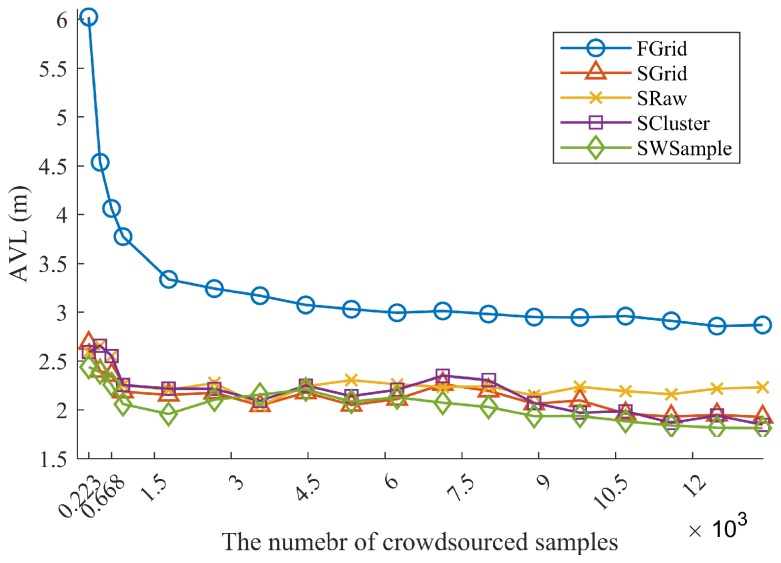
Comparison of localization performance, when using crowdourced samples only from $S_{walk}$. The average localization error (ALE) vs. the number of crowdsourced samples $M_{all}$, where σ=1.2 m.

**Figure 12:**
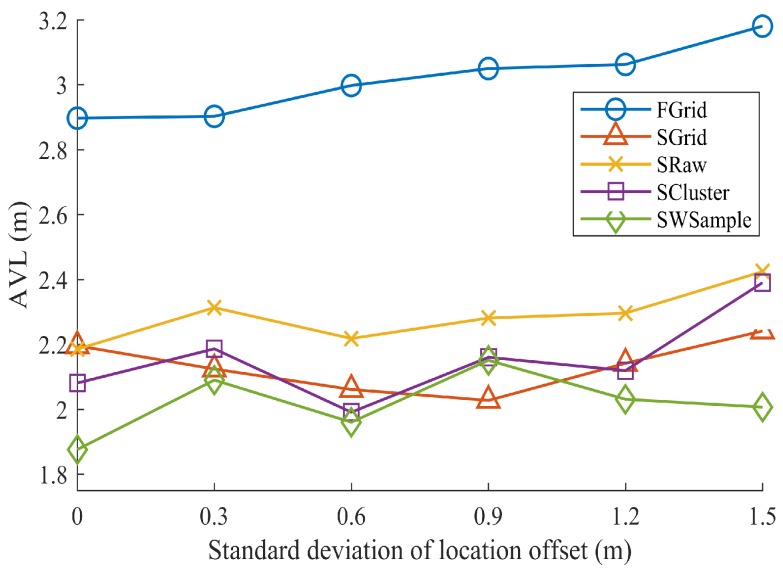
Comparison of localization performance, when using crowdsourced samples only from $S_{walk}$. The average localization error (ALE) vs. the standard deviation σ of location offset, where $M_{all}=4456$.

**Figure 13:**
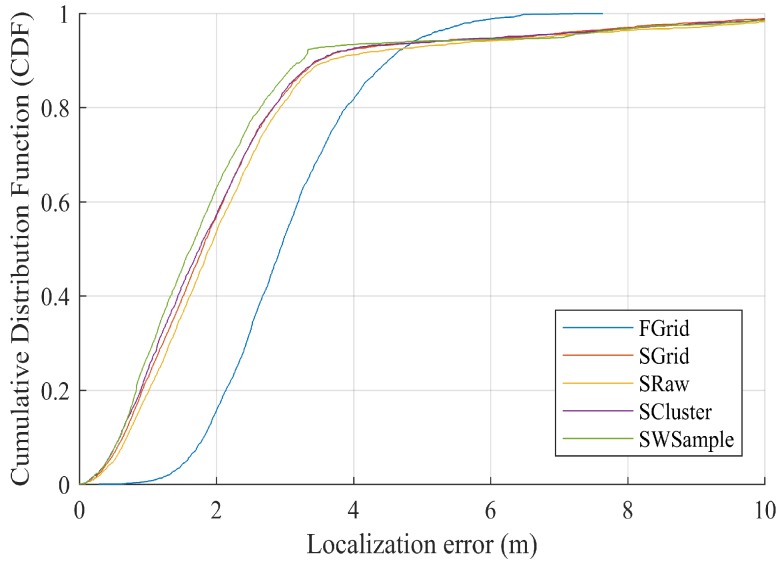
Comparison of cumulative distribution function (CDF) localization error with $M_{all}=4456$ and σ=1.2 m, when using crowdourced samples only from $S_{walk}$.

**Non-uniformly distributed samples:** It is also often the case that crowdsourced samples are not uniformly distributed in the whole environment. To examine this *nonuniform density* issue, we only use the samples from $S_{walk}$ to fit surfaces. That is, the subregion of chairs and desks in each room do not contain crowdsourced samples. However, as we intentionally include location annotation errors, some samples may still be annotated to locations within such a vacant subregion. As shown in Figure 11 and Figure 12, it is not unexpected to observe that all schemes suffer from such a nonuniform density situation, comparing with the results in Figure 8. However, our SWSample scheme can still outperform other schemes in most of cases. The positioning accuracy improvements are 36.85% over FGrid and 18.79% over SRaw, respectively. Furthermore, the median and 90% localization errors in Figure 13 are 2.04 m and 3.24 m, respectively, which are comparable to the uniform density case. Table 2 summarizes the localization error results from three situations for non-uniformly distributed samples. It can be observed that our proposed scheme has great potential to obtain a better result in this non-uniformly distributed case, which illustrates its robustness for tackling the nonuniform density challenge.

## 6. Concluding Remarks

This paper has studied the problem of constructing radio propagation surfaces from unreliable crowdsourced samples with annotation errors. We have proposed a cross-domain cluster intersection to weight each sample reliability and an entropy-like approach to further weight the constructed surfaces. Field experiments have validated its effectiveness and robustness for dealing with the nonuniform density challenge. Our proposed method contributes to indoor localization society in its high accuracy and easy implementation.

We close this paper with some discussions about future work. This paper has applied polynomial functions for fitting radio propagation surfaces in the offline phase. Indeed, the propagation surfaces may take different forms and there could exist many other primary functions or stochastic kernels for surface fitting. How to intelligently choose the most suitable primary functions or stochastic kernels and automatically adjust their fitting parameters are worthy of further research. In this paper, we have applied the commonly used deterministic positioning algorithm in the online phase. Using some probabilistic positioning algorithms, especially when the radio propagation surfaces are modelled as stochastic processes, is also worthy of further investigation.

## Figures and Tables

**Table 1:** Table of symbols.

Symbol	Definition
S	A set of crowdsourced samples in one subarea.
*M*	The number of crowdsourced samples in S, M=|S|.
$s_{i}$	The *i*th crowdsourced sample in S.
${\vec{l}}_{i}$	The annotated location of the *i*th crowdsourced sample.
${\vec{r}}_{i}$	The RSS vector of the *i*th crowdsourced sample.
*N*	The maximum number of hearable AP in S.
*K*	The number of clusters.
$C^{p}$	The set of clusters in the physical space.
$C^{s}$	The set of clusters in the signal space.
$\gamma_{i}$	The cross-domain cluster coefficient of the *i*th sample.
$\omega_{i}$	The reliability weight of the *i*th sample.
ϕ(x,y)	The RSS surface function.
$p_{th}$	The percentile threshold in sample selection method.
$\omega_{th}$	The weight threshold in sample selection method.
$\vec{\omega }$	The increasing order of sample reliability weight.
$\omega_{k}$	The reliability weight at the $p_{th}$ percentile in $\vec{\omega }$.
$S^{\prime }$	The set of select samples.
A	The set of hearable Aps by samples in $S^{\prime }$.
$\alpha_{ij}$	The surface coefficient of the RSS surface function.
R	The set of RSS values from an AP in $S^{\prime }$.
${\bar{r}}_{i}$	The normalized elements in R.
η	The entropy-like quantity for each AP in A.
$\rho_{n}^{sub}$	The surface weight of *n*th AP in A for subarea determination.
$\rho_{n}^{loc}$	The surface weight of *n*th AP in A for location search.
$\vec{f}$	Subarea fingerprint.
G	The set of grid cells in one subarea.
*G*	The number of grids in G, G=|G|.
${\vec{f}}_{t}$	The RSS vector of a test sample.
${\vec{f}}_{s}$	The *s*th subarea fingerprint.
$A_{int}$	The set of hearable APs by both ${\vec{f}}_{t}$ and ${\vec{f}}_{s}$.
$D_{s}$	The weighted signal distance between the test sample and a subarea.
$M_{g}$	The number of grid cells.
σ	The standard deviation of location offset.
$S_{site}$	The set of samples from site survey.
$S_{walk}$	The set of samples from pedestrian trajectories.

**Table 2:** Comparison of mean, 50% and 90% localization error.

Error (m)		σ=0 m			σ=0.6 m			σ=1.2 m		
		Mean	50%	90%	Mean	50%	90%	Mean	50%	90%
Uni.	FGrid	2.479	2.448	3.672	2.284	2.086	3.744	2.421	2.217	3.868
	SGrid	1.571	1.353	2.595	1.726	1.630	2.884	1.898	1.757	3.048
	SRaw	1.575	1.370	2.645	1.618	1.524	2.694	1.711	1.688	2.873
	SCluster	1.552	1.364	2.550	1.708	1.657	2.875	1.916	1.879	3.111
	SWSample	1.373	1.124	2.413	1.374	1.243	2.470	1.513	1.366	2.640
Non-uni.	FGrid	2.897	2.776	3.672	2.982	2.813	4.477	3.059	2.932	4.502
	SGrid	2.164	1.691	3.522	2.086	1.679	3.402	2.169	1.795	3.499
	SRaw	2.155	1.713	3.459	2.221	1.732	3.594	2.322	1.898	3.647
	SCluster	2.063	1.602	3.497	2.009	1.584	3.287	2.144	1.752	3.477
	SWSample	1.854	1.497	3.172	1.951	1.472	3.217	2.043	1.625	3.242
